# Ageing-resembling phenotype of long-term allogeneic hematopoietic cells recipients compared to their donors

**DOI:** 10.1186/s12979-022-00308-6

**Published:** 2022-11-02

**Authors:** Michał Cezary Czarnogórski, Justyna Sakowska, Mateusz Maziewski, Maciej Zieliński, Agnieszka Piekarska, Igor Obuchowski, Mikołaj Młyński, Magdalena Dutka, Alicja Sadowska-Klasa, Ewa Zarzycka, Maria Bieniaszewska, Piotr Trzonkowski, Jacek M. Witkowski, Andrzej Hellmann, Katarzyna Ruckemann-Dziurdzińska, Jan M. Zaucha

**Affiliations:** 1 Department of Hematology and Transplantology, Medical University of Gdańsk, Gdańsk, Poland; 2 Department of Physiopathology, Medical University of Gdańsk, Gdańsk, Poland; 3 Department of Medical Immunology, Medical University of Gdańsk, Gdańsk, Poland; 4 Intercollegiate Faculty of Biotechnology, Medical University of Gdańsk, University of Gdańsk, Gdańsk, Poland; 5 Department of Pathology and Experimental Rheumatology, Medical University of Gdańsk, Gdańsk, Poland

**Keywords:** allo-HCT, Ageing, Immunosenescence, Telomeric shortening

## Abstract

**Background:**

Ageing is a complex phenomenon that leads to decreased proliferative activity, loss of function of the cells, and cellular senescence. Senescence of the immune system exacerbates individual’s immune response, both humoral and cellular but increases the frequency of infections. We hypothesized that physiological ageing of adaptive immune system occurs in recipients of allogeneic hematopoietic cells transplant (allo-HCT) at faster rate when compared to their respective donors since the small number of donor cells undergo immense proliferative stress restoring recipients hematopoiesis. We compared molecular characterizations of ageing between recipients and donors of allo-HCT: telomeric length and immunophenotypic changes in main lymphocyte subsets – CD4^+^, CD8^+^, CD19^+^, CD56^+^.

**Results:**

Median telomeric length (TL) of CD8^+^ lymphocytes was significantly longer in donors compared to recipients (on average 2,1 kb and 1,7 kb respectively, p = 0,02). Similar trends were observed for CD4^+^ and CD19^+^ although the results did not reach statistical significance. We have also found trends in the immunophenotype between recipients and donors in the subpopulations of CD4^+^ (naïve and effector memory), CD8^+^ Eomes^+^ and B-lymphocytes (B1 and B2). Lower infection risk recipients had also a significantly greater percentage of NK cells (22,3%) than high-risk patients (9,3%) p = 0,04.

**Conclusion:**

Our data do not support the initial hypothesis of accelerated aging in the long term all-HCT recipients with the exception of the recipients lymphocytes (mainly CD8^+^) which present some molecular features, characteristic for physiological ageing (telomeric shortening, immunophenotype) when compared to their respective donors. However, a history of lower infection numbers in HCT recipients seems to be associated with increased percentage of NK cells. The history of GVHD seems not to affect the rate of ageing. Therefore, it is safe to conclude that the observed subtle differences between recipients’ and donors’ cells result mainly from the proliferative stress in the early period after allo-HCT and the difference between hosts’ and recipients’ microenvironments.

**Supplementary information:**

The online version contains supplementary material available at 10.1186/s12979-022-00308-6.

## Background

Ageing involves a series of biological processes that lead to gradual loss or change in the function of body cells. Although many questions remain unanswered, some molecular mechanisms of ageing have already been identified. They include telomeric shortening and age-associated changes in immunophenotype.

Telomeric shortening occurs with every cellular division. After reaching the critical length of telomeres a cell enters the senescent phase or undergoes apoptosis [1]. The most pronounced telomeric shortening throughout a life happens in lymphocytes. At birth mean telomeric length for lymphocytes is ~ 11 kb and decreases to ~ 4 kb at the age of 100 years. With ageing, telomeric shortening gradually decelerates. The average annual rate of telomeric shortening for human lymphocytes is 1190 bp in the first year of life, than 126 bp/year in childhood and 43 bp/year through the rest of adult life [2].

Immunophenotypic changes associated with ageing include, among others, an increase in the proportion of anergic CD8^+^ lymphocytes leading to a decreased ratio of CD4^+^/CD8^+^ lymphocytes, an increased proportion of Treg and Th2 lymphocytes, and loss of CD28. [3, 4]. CD28^−^ T-cells are characterized by reduced replicative lifespan and decreased proliferative capacity, as well as by reduced response for antigen stimulation while exhibiting increased cytotoxic activity [5].

The key aspect of allogeneic hematopoietic cells transplantation (allo-HCT) is the restoration of the whole hematopoiesis in the recipient from the relatively small 2–5 × 106/kg number of donor stem cells. Thus, the transplanted cells are exposed to immense proliferative stress compared to identical cells that remain in the donor system. [6]. The immune part of the hematopoietic system is particularly exposed to the proliferative stress since it is also stimulated by the differences between recipient’s and donor’s minor histocompatibility antigens (MiHAs) leading to the graft versus host reaction which clinically is manifested as graft versus host disease (GvHD) [7]. Moreover, recipients of allo-HCT are susceptible to infectious complications that cause additional proliferative stress to immune cells [8]. Consequently, we have hypothesized that progeny of the donor HSC in the recipients of allo-HCT undergoes accelerated ageing, which may be responsible for those clinical consequences.

Thus, in our study we compared (1) the magnitude of telomeric shortening of the transplanted donor cells subpopulation to the same cells subpopulations that remained intact in the donor (2) immunophenotypic changes of respective lymphocyte subpopulations between donors and their respective recipients.

## Methods

### Patients

We enrolled 20 pairs of donors (D) and their related recipients (R) undergoing the allo-HCT at least more than 12 years ago (long-term survivors) at the University Clinical Center, Medical University of Gdańsk, Gdańsk, Poland (EBMT accredited center 799). The number of pairs [20] was limited by overall mortality related to the procedure and availability of long-term survivors. For every recipient-donor pair sample of 50ml of full venous blood were collected with anticoagulant (EDTA), at single timepoint.

### GvHD and infectious status assessment

Patients were stratified according to chronic GvHD status (Yes versus No) and infectious complications according to an infection risk status score (Table 1.) that was based on the number of infections in the last year and the need for antibiotic usage or hospitalization.

### Peripheral blood mononuclear cells (PBMC) and lymphocyte isolation

PBMC was obtained from venous blood and centrifugation over a Ficoll-Hypaque (Ficoll-Paque PLUS assay (GE Healthcare, USA) gradient. Lymphocytes were isolated from PBMC by immunomagnetic positive separation technique with magnetic particles (EasySep Kit III from STEMCELL™ Technologies) recognizing respective CD4^+^, CD8+, CD19 + or CD56 + antigens. The purity of each cell population was > 90% (assessed by flow cytometry), sufficient for further parts of the experiment. [4, 9] Isolated lymphocyte subpopulations were pelleted by centrifugation and stored at -80^o^C for further processing.

**Table 1:** Infection risk status

	Infection risk status		
**No of episodes of infections (during last year)**		**SCORE**	
	**Without antibiotic**	**With antibiotic**	**Hospitalization**
**0**	0	0	0
**1**	1	2	3
**2**	2	4	6
**≥3**	3	6	9
			**TOTAL**
	**Low risk**		< 3
	**High risk**		>=3

### Telomeric length measurement

**Fig. 1:**
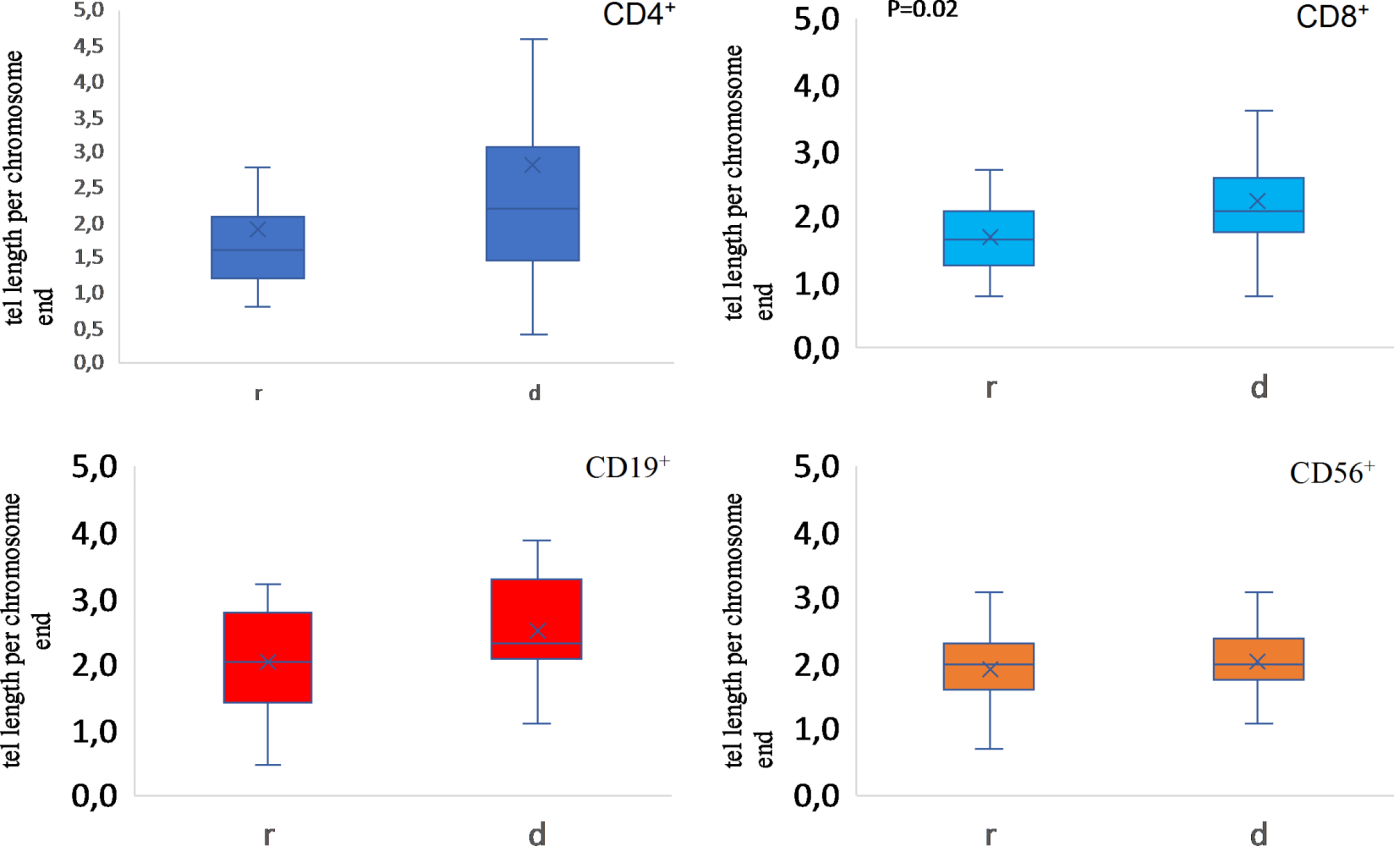
Box plots of mean telomeric length (median kb) in recipients (R) of allo-HCT and their donors (D) in main lymphocyte subpopulations CD4^+^, CD8^+^, CD19^+^ and CD56^+^. The box and whiskers plots are corresponding to median, 25th and 75th quartile and outlayers. Means are marked as X

Determination of the average telomeric length was performed using quantitative polymerase chain reaction (qPCR) applying the commercially available Absolute Human Telomere Length Quantification qPCR Assay Kit (from ScienCell Research Laboratories). The single-copy reference primers (included in the kit), recognizing and amplifying a 100 bp sequence of chromosome 17 were used as a reference for data normalization. The reference DNA sample with established telomere length (also included in the kit) served as a reference for the assessment of the telomeric length. Acquired results for every sample were then computed according to the manufacturer’s instructions. The total length of all telomere ends in a single cell was divided by the number of telomeric ends (92) which is the final result shown in the Fig. 1. The final result is a median of two independent measurements per individual sample.

### Immunophenotyping

Stored lymphocytes obtained as above were thawed and their viability was checked with trypan blue assay using TC20 Automated Cell Counter (Bio-Rad Laboratories, USA. The viability cut-off was set to 80%. Next, samples of 2 × 10^5^ cells were stained with anti-CD45 (clone HI30), anti-CD3 (clone OKT3), anti-CD4 (clone MEM-241), anti-CD19 (clone HIB19), anti-CD5 (clone UCHT2), (all from Thermo Fisher Scientific, USA) and anti-CD8 (clone RPA-T8), anti-CD56 (clone NCAM16.2) (all from BD Bioscience, USA). For intracellular staining anti-Foxp3 (clone PCH101), and anti-Helios (clone 22F6) were used with Foxp3 / Transcription Factor Staining Buffer Set (all from Thermo Fisher Scientific, USA). Samples were read out with LSRFortessa flow cytometer (BD Bioscience, USA) and for every sample, a minimum of 75.000 events was recorded.

Flow cytometry data were analyzed with Kaluza 1.2 software (Beckman Coulter, USA). First, doublets were excluded by FSC area (FSC-A) and FSC height (FSC-H) discrimination, and then lymphocytes were identified upon SSC/CD45^+^ gating. Major lymphocytes subsets were identified as: lymphocytes T, both CD3^+^/CD4^+^, and CD3^+^/CD8^+^, lymphocytes B, CD19^+^, NK cells CD3^−^/CD56^+^, B1 B cells, CD5^+^/CD19^+^, B2 B cells, CD5^−^/CD19^+^, and regulatory T cells, Foxp3^+^/CD4^+^/CD3^+^. Gating was done upon FMO (fluorescence minus one) approach. The absolute count of CD4^+^ and CD8^+^ was calculated using a percentage of CD4^+^ and CD8^+^ from immunophenotyping and absolute lymphocyte count (ALC) obtained from Sysmex hematology analyzer.

### Statistical analysis

All statistical calculations were performed using the StatSoft Inc. 2014 – STATISTICA version 12.0 (www.statsoft.com) and Microsoft Excel spreadsheet. Quantitative variables were characterized by the arithmetic mean, standard deviation, median, minimum and maximum (range), and 95%CI (confidence interval). Qualitative variables were displayed by number and percentage unless noted otherwise. For testing, if the quantitative variable was derived from the population with the normal distribution, the W Shapiro-Wilk test was selected. For testing the hypothesis of equal variances, the Leven’s (Brown-Forsythe) test was used. Significance of differences between two groups (independent samples model) was tested by Student’s t-test (in case of lack of homogeneity of variance – Welch t-test) or by U Mann-Whitney test (in case of not fulfilling the conditions to use the Student’s t-test or for ordinal variables). The significance of differences between more than two groups was verified using Kruskal-Wallis test. In the case of receiving statistically significant differences between groups, Dunn test was performed. Data were visualized using box and whiskers plot displays. The confidence interval (CI) of 95% was preconceived, p value < 0.05 was considered significant.

## Results

Patient characteristics is summarized in Table 2. The median time from HCT was 17,4 (range 12 to 25) years. Twelve male and 8 female recipients received allo-HCT due to a variety of hematological disorders (Table 2). Eight (40%) recipients had a history of chronic GvHD. None of those recipients required active immunosuppressive treatment at the time of study enrollment. Infectious status was low in 12 recipients whereas the rest had high risk [8] infectious status according to our infectious risk stratification model (Table 1).

**Table 2:** Patients’ characteristics

Patient no.	Diagnosis	Sex (R/D)	Time since allo-HCT (years)	Age at allo-HCT (years) R/D	Conditioning regimen	Chronic GvHD *	Infection risk status (low, high) **	Number of CD34^+^cells infused (x 10^6/kg)
1	CML	M/F	25	33/27	BuCy	-	Low	-
2	ALL	F/M	18	20/15	TBI	-	High	7,78
3	AML	M/M	15	23/25	BuCy	-	Low	6,3
4	AML	F/M	20	36/46	BuCy	Yes	Low	11,6
5	HES	M/F	19	32/33	BuCy	-	Low	1,56
6	CML	M/M	18	46/43	BuCy	-	Low	5,6
7	CML	F/F	17	22/10	BuCy	Yes	Low	-
8	PNH	M/M	18	27/20	BuCy	-	Low	1,31
9	CML	M/F	23	39/41	BuCy	Yes	High	-
10	AML	M/F	14	43/39	BuCy	Yes	High	-
11	AML	F/F	17	47/43	BuCy	Yes	High	-
12	CML	M/M	19	36/18	BuCy	-	High	3,4
13	ALL	F/M	24	28/24	BuCy	-	Low	-
14	AML	M/F	15	31/28	BuCy	-	Low	3,9
15	CML	M/M	20	44/43	BuCy	-	Low	8
16	MDS	F/F	12	42/43	BuCy	Yes	High	1,94
17	CML	F/M	17	38/43	BuCy	-	High	1,35
18	AML	F/M	12	38/38	BuCy	Yes	Low	6,06
19	CML	M/M	13	33/22	BuCy	Yes	High	-
20	AML	M/F	12	41/54	BuCy	-	Low	0,88

* History of chronic cGvHD

** Status assessment according to Table 1

(CML – chronic myelogenous leukemia, ALL – acute lymphoblastic leukemia, AML – acute myelogenous leukemia, HES – hypereosinophilic syndrome, PNH – paroxysmal nocturnal hemoglobinuria, MDS – myelodysplastic syndrome, R – recipient, D – donor, MRD – matched related donor, MUD – matched unrelated donor, BuCy – busulfan & cyclophosphamide, TBI – total body irradiation)

## Results

### Pairwise (recipients vs. donors) comparison of telomeric length

Median of telomeric length, expressed in kb per chromosome end) in CD8^+^ lymphocytes was significantly greater in D (2,1 kb [95%CI 1,8;2,7]) compared to R (1,7 kb [95%CI 1,4;1,9]) (p = 0,02; n = 40). There were also similar tendencies in CD4^+^ and CD19^+^ lymphocyte subpopulations, respectively D – 2,2 kb [95%CI 1,8;3,8], R – 1,6 kb [95%CI 1,4;2,4] (p = 0,1; n = 40) and D – 2,3 kb [95%CI 2,1;2,9], R- 2,1 kb [95%CI 1,7;2,4] (p = 0,076; n = 40), although they have not reached statistical significance. We have not found differences in the CD56^+^ population (D – 2 kb [95%CI 1,8;2,3], R – 2 kb [95%CI 1,5;2,3] (p = 0,53) (n = 40)) (Fig. 1.). We have checked the influence of gender and age of the donors on the mean telomere length in recipients and have found no correlation. In the CD8^+^ cells population the age of donors was inversely correlated with mean telomere length of the donors (Correlation coefficient − 0.59; p = 0.007; Spearman). Also, the number of CD34^+^ cells infused was inversely correlated with mean telomeric length of the CD8^+^ cells of recipients (Correlation coefficient − 0.55; p = 0.05; Spearman) (Table Suppl 9–18).

### Immunophenotype analysis

Median percentage of T CD4 + was significantly greater in D than in R: 44,3% (95%CI 37,2;48,3) and 40,1% (95%CI 31,9;40,8) respectively (p = 0,05; n = 34). In contrast CD19^+^ percentage was greater in R than in D: mean 11,3% (95%CI 9,8;13,5) and 8,5% (95%CI 7,8;11,9) respectively (p = 0,03; n = 34). (Table 3) Moreover we observed difference trends in few others lymphocyte subpopulations (p value approaching 0.05, Table 3). Among the population of CD4^+^ there was greater percentage of effector memory (CD4^+^ EM) cells in R than D: 28,8% (95%CI 23,4;37,5) and 19,8% (95%CI 16,5;27,8) (p = 0,07; n = 34) respectively and lower percentage of CD4^+^ naïve cells in R than D: 24,5% (95%CI 16,9;33,8) and 38% (95%CI 28,8;43,5) (p = 0,06 n = 34) respectively. Among the CD8^+^ subpopulation there was greater percentage of CD8^+^ expressing eomesodermin (CD8^+^ Eomes) in R – 39,4% (95%CI 29,7;47,7) than D – 31,5% (95%CI 24,2;36,7) (p = 0,07; n = 34). Among the CD19 + population there was greater percentage of B1 lymphocytes in D – 21,7% (95%CI 17;27,5) than R – 17,2% (95%CI 12,8;24,3) (p = 0,08; n = 34) and greater percentage of B2 lymphocytes in R – 81,6% (95%CI 74,4;86,4) than D – 77% (95%CI 71,1;82) ( p = 0,07; n = 34) ) (Table 3).

**Table 3:** Immunophenotypic differences between recipients and donors of allo-HCT

	R (n = 17)	D (n = 17)	***P***-value
**CD4** ^**+**^			**0.05**
mean (SD)	36,4 (8,4)	42,8 (10,4)	
range	19,9–49,5	16,8–58,4	
median	40,1	44,3	
95%CI	[31,9;40,8]	[37,2;48,3]	
**CD4** ^**+**^ **Effector Memory**			0.07
mean (SD)	30,4 (13,2)	22,2 (10,7)	
range	13,0–59,0	9,0–54,1	
median	28,8	19,8	
95%CI	[23,4;37,5]	[16,5;27,8]	
**CD4** ^**+**^ **Naive**			0.06
mean (SD)	25,3 (15,9)	36,1 (13,8)	
range	4,6–55,3	2,4–52,9	
median	24,5	38,0	
95%CI	[16,9;33,8]	[28,8;43,5]	
**CD8** ^**+**^ **Eomes**			0.07
mean (SD)	38,7 (16,3)	30,4 (11,2)	
range	1,3–66,9	11,1–49,5	
median	39,4	31,5	
95%CI	[29,7;47,7]	[24,2;36,7]	
**CD19** ^**+**^			**0.03**
mean (SD)	11,7 (3,4)	9,8 (3,9)	
range	7,4–20,0	5,9–19,5	
median	11,3	8,5	
95%CI	[9,8;13,5]	[7,8;11,9]	
**B1**			0.08
mean (SD)	18,5 (10,8)	22,2 (9,8)	
range	2,6–49,8	5,7–47,4	
median	17,2	21,7	
95%CI	[12,8;24,3]	[17,0;27,5]	
**B2**			0.07
mean (SD)	80,4 (11,2)	76,5 (10,2)	
range	48,1–97,2	50,4–93,7	
median	81,6	77,0	
95%CI	[74,4;86,4]	[71,1;82,0]	

## CD4^+^/CD8^+^ ratio

Median CD4^+^/CD8^+^ ratio was higher in donors than in recipients of allo-HCT – 2,1 (95%CI 1,3;2,1) and 1,5 (95%CI 1,8;2,6) respectively (p = 0,0396) (n = 38) (Table 4).

**Table 4:** CD4^+^/CD8^+^ ratio in recipients of allo-HCT and their donors

	R (n = 19)	D (n = 19)	***P***-value (U Mann-Whitney)
**CD4** ^**+**^ **to CD8** ^**+**^			**0,0396**
mean (SD)	1,7 (0,9)	2,2 (0,9)	
range	0,7 − 4,6	1,0–4,6	
median	1,5	2,1	
95%CI	[1,3;2,1]	[1,8;2,6]	

^1^U Mann-Whitney

## Analysis of the recipients of allo-HCT depending on the infection status

### Immunophenotype analysis

Differences in immunophenotype were also tested in recipients divided again into two groups: low risk and high risk of infection. We have found significant differences in the percentage of NK cells (CD56^+^), which was higher in low risk recipients’ group (p = 0,0344). Furthermore, among the NK cells population we have found differences in the NK cells with the expression of perforin (NK Perforin) and CD28. NK Perforin percentage was higher in low risk recipients group (p = 0,0079) and NK CD28^+^ percentage was higher in high risk patients group. There was also a difference in the percentage of NK dim cells – it was higher in low risk recipients group (p = 0,0344) (Table 5).

**Table 5:** Immunophenotype comparison between recipients of allo-HCT grouped according to infection risk status

Parameter	Low risk	High Risk	***P***-value
%NK Perforin^+^			**0,0079**
mean (SD)	86.4 (29.8)	57.9 (44.0)	
range	2.2–99.8	1.4–92.3	
median	95.2	82.0	
95%CI	[65.1;107.7]	[11.7;104.1]	
%NK CD28^+^			**0,0344**
mean (SD)	6.5 (8.7)	14.3 (9.5)	
range	1.8–30.8	3.6–27.5	
median	3.8	11.1	
95%CI	[0.3;12.7]	[4.4;24.3]	
%NK CD56^dim^			**0,0433**
mean (SD)	18.5 (12.6)	6.5 (5.8)	
range	0.1–45.9	0.1–15.4	
median	20.0	6.9	
95%CI	[9.5;27.5]	[0.4;12.6]	
%NK			**0,0448**
mean (SD)	22.1 (13.0)	10.5 (3.1)	
range	9.3–52.0	7.1–15.3	
median	22.3	9.6	
95%CI	[12.8;31.4]	[6.6;14.3]	

## Discussion

In our work, we assumed that studying long-term surviving donor-recipient allo-HCT pairs would allow us to find differences between the donors’ transplanted cells exposed to immense proliferative and environmental stress which accelerated their ageing and the donor cells that remained intact in the donor and were ageing naturally. Such a scenario limits the number of major factors affecting the differences in ageing between donors and recipients’ lymphocyte populations to just two: allogeneic transplantation itself and the different host’s microenvironments. We have tested TL in four main lymphocyte subpopulations and found that the telomeres were significantly shorter (0,4 kb) in the T CD8^+^ lymphocyte subpopulation of the recipients. The similar tendencies have been found for T CD4^+^ and B (CD19^+^) lymphocytes – telomeres were shorter in recipients by 0,6 kb (p = 0,1) and 0,2 kb (p = 0,076) respectively. The strong difference between recipients and donors in CD8^+^ population may result from faster reconstitution of CD8^+^ lymphocytes population in the recipient compared to CD4^+^ population after allo-HCT [10–12]. Moreover, the increased proliferation of CD8^+^ corresponds well with the inverted CD4^+^/CD8^+^ ratio in recipients of allo-HCT which is observed at least in the first 2 years after transplantation [12, 13]. The lack of any difference nor any trend for the difference in TL in NK cells (CD56^+^) is difficult to explain. Our observation might be partially explained by the fact that NK cells are the first to proliferate during the reconstitution period and may reach the normal values even within a month after allo - HCT [14, 15]. This could lead to relatively small proliferative stress and in consequence, would be reflected by lack of significant telomeric length shortening. Moreover, it is unlikely that increased endogenous telomerase activity is responsible for this observation because of the low telomerase activity in aged NK cells [16]. The telomere length did not differ between recipients and donors respectively depending on the age of the recipients or gender. We also have not found differences in the mean telomere length of the recipients in any lymphocyte subpopulation tested depending on the donor’s age (Table Suppl 17.). Interestingly, the analysis has shown inverse correlation of mean telomere length of donors and age of donors but only in CD8^+^ lymphocyte subpopulation. Those findings seem to confirm crucial impact of allo-HCT on telomeric shortening since the inverse correlation of age of donor and donor’s mean telomeric length was not observed in recipient’s mean telomeric length in the same lymphocyte subpopulation tested (CD 8^+^). Strangely, the mean telomere length of the recipients was also inversely correlated with the number of cells transplanted and also only in CD8^+^ lymphocyte subpopulation (Tables 16 and 17 in Supplementary Material). However, interpretation of this result is challenging since stem cells only consist of some percentage (different in each donor) of CD34^+^ cells. The other conceivable factor (not studied in the work) that may influence the results may be the telomerase activity of the stem cells.

We have found differences in the median percentage of CD4^+^ lymphocytes – it was higher in donors (44,3%) than in recipients (40,1%). Among CD4^+^ population there were also similar tendencies in CD4^+^ naïve cells and CD4^+^ EM (Effector Memory) cells. CD4^+^ naïve cells accounted for 24,5% in recipients and 38% in donors (p = 0,06). On the other hand CD4^+^ EM comprised of 28,8% in recipients and 19,8% in donors (p = 0,07). Interestingly, such changes are typical for physiological ageing. Physiologically, the decrement of naïve cells during the ageing process is caused mainly due to thymic involution, as well as expansion of memory cells [17]. In the allo-HCT long-term survivors, the mechanism could be similar as thymus suffers considerable injury after conditioning [18]. Though the decrement of the percentage of naïve cells is not limited to CD4^+^ naïve cells, we did not find differences nor any trends in CD8^+^ naïve cells. Moreover, with age the percentage of differentiated CD4^+^ and CD8^+^ memory and central memory cells increases [19]. Though we have found such tendency in CD4^+^ EM, strangely there were no trends in CD8^+^ EM. The increased proliferation of CD8^+^ lymphocytes was already mentioned above. We did not find differences or trends in CD8^+^ percentages with the exception found in the subpopulation expressing Eomesodermin (CD8^+^ Eomes^+^). In recipients it was greater than in donors – 39,4% and 31,5% respectively (p = 0,07). Eomesodermin is a transcription factor expression of which in CD4^+^ and CD8^+^ seems to be essential for development of effector memory cells [20] and therefore increased percentage of CD4^+^ and CD8^+^ with expression of this transcription factor may be one of the indicators of aged immune system.

In our study, we have found a significant decrease in CD4^+^/CD8^+^ ratio in recipients [1, 5] compared to their donors [1, 2] who retained normal CD4^+^/CD8^+^ ratio [21] (Table 4). Interestingly, in physiological ageing, inverted CD4^+^/CD8^+^ is common. It affects about 16% of people between 60 and 94 years of age [22] and is one of the features of immunosenescence [23, 24]. Our observation may suggest that decreased CD4^+^/CD8^+^ ratio in allo-HCT recipients is a sign of T cell exhaustion and/or accelareted ageing induced by allo-HCT.

We have found that B-cell percentage of the total lymphocyte population significantly differs between recipients and donors – 11,3% and 8,5% respectively (p = 0,03). In physiological ageing, we observe a decrement of both percentage and absolute count of CD19^+^ cells [25, 26]. Strangely, we have found an increased percentage of B-lymphocytes in recipients compared to their donors. This might result from the increased incidence of autoimmune diseases in all-HCT recipients compared to their respective donors as an example of “alloimmunization” [27]. We also observed some interesting trends in the percentages of B1 and B2 lymphocytes. Recipients tended to show lower percentage of B1 lymphocytes – 17,2% compared to donors 21,7% (p = 0,08) and greater percentage of B2 lymphocytes 81,6% in recipients compared to 77% in donors (p = 0,07). In physiological ageing, the proportion of B1 cells which produce antibodies without antigen stimulation and are the part of innate immunity [28] decreases with age which may be connected with increased incidence of infections in older age [29]. As a consequence, the proportion of B2 cells which make up the majority of B-cells is increased though the absolute count decreases [26]. It seems that changes in B-cells in recipients of allo-HCT tend to mimic those observed in physiological ageing process.

## Infectious risk status influence

We were not able to confirm our initial hypothesis of greater telomeric shortening in individuals with high infectious risk status. This observation supports Mathioudakis et al. suggestion that demand for increased proliferation of hematopoietic stem cells stabilizes early after the period of initial post-transplant acceleration [30] and maybe limited only to the reconstitution period and is not affected by other post-transplant complications.

Immunophenotypic differences between recipients stratified according to infection risk status revealed that in recipients with low risk status there were higher percentages of NK cells (p = 0,0344). Among NK cells there were also higher percentage of NK ^dim^ population (p = 0,0344) and NK with the expression of perforin NK Perforin (p = 0,0079) in recipients with low risk status. In physiological ageing process there is an increase in NK cells percentage and among them most pronounced in NK^dim^ population [31, 32]. Interestingly, the perforin (that is an effector of the cytotoxic activity of those cells) expression declines with age [32]. It would suggest that about 60% of recipients with lower incidence of infections (Table 1.) present both features of the aged innate immune system (NK cells specifically) and increased cytotoxic activity (increased Perforin expression) which results in decreased incidence of infections. We did not find any differences or even trends in Treg or NK cells populations though in physiological ageing process the number of Tregs decreases [33] and NK cells, especially dim population increases [31]. However, our sample could have been too small to identify them. Interestingly, changes in NK cells in low-risk status recipients may imply the pivotal role of the innate immune system in protection against infections in recipients of allo-HCT.

To our surprise, the history of GVHD did not affect any studied outcomes It may be due to multiple factors – history of pharmacological immune suppression, resolution of all GVHD symptoms at the time of entering our study, the presence of age-related diseases, and finally small sample size.

There are some limitations of our study- the most important is a bias regarding donor-recipient pair selection based on the long-term survival of the whole pair. An additional important limitation is the lack of information on the aging status of the hematopoietic system of the donors at the time of donation which obviously is not accessible anymore.

## Conclusion

To conclude, our findings would suggest that recipients’ lymphocytes seem to have some features of physiological ageing when compared to their respective donors which is reflected by the difference in the telomere length (mainly CD8 subset) and immunophenotypic quantitative changes of transplanted cells, characteristic for ageing. However, a history of lower infection numbers in HCT recipients seems to be associated with an increased percentage of NK cells. The history of GVHD does not affect the rate of ageing. Therefore, the observed differences between transplanted and not transplanted cells most likely result from the huge proliferative stress in the early period after allo-HCT and to some extent the difference between host and recipients’ microenvironments which is the only other variable that may influence the identical cells originating from donor hematopoiesis.

## Electronic supplementary material

Below is the link to the electronic supplementary material.

Supplementary Material 1

## Data Availability

The dataset supporting the conclusions of this article are included within the article and supplementary material.
