# Supplementary material for: Ageing-resembling phenotype of long-term allogeneic hematopoietic cells recipients compared to their donors
Source: Immun Ageing. 2022 Nov 2;19:51. doi: 10.1186/s12979-022-00308-6 (PMC9628063; doi:10.1186/s12979-022-00308-6)
Supplement: Supplementary file 1 — Supplementary Material 1 [file 12979_2022_308_MOESM1_ESM.docx]

Table Suppl 1. Comparative characteristics of telomeric length in CD4 subpopulations in recipients grouped according to infection status.

|  | **Low risk**  **(n=12)** | **High risk**  **(n=8)** | ***P*-value** |
| --- | --- | --- | --- |
| **tel length** |  |  | 0,1325^1^ |
| mean (SD) | 203,4 (125,1) | 134,9 (41,5) |  |
| range | 74,0-516,8 | 98,0-219,9 |  |
| median | 168,0 | 119,0 |  |
| 95%CI | [123,9;282,9] | [100,2;169,6] |  |
| tel lenght +/- |  |  | 0,2318^1^ |
| mean (SD) | 12,7 (10,4) | 7,2 (3,6) |  |
| range | 2,9-37,5 | 2,3-12,1 |  |
| median | 9,6 | 7,8 |  |
| 95%CI | [6,1;19,3] | [4,2;10,2] |  |
| tel per chromosome |  |  | 0,1427^1^ |
| mean (SD) | 2,2 (1,3) | 1,5 (0,4) |  |
| range | 0,8-5,6 | 1,1-2,4 |  |
| median | 1,8 | 1,3 |  |
| 95%CI | [1,4;3,1] | [1,1;1,9] |  |
| tel +/- per chromosome |  |  | 0,3749^1^ |
| mean (SD) | 0,1 (0,1) | 0,1 (0,0) |  |
| range | 0,0-0,4 | 0,0-0,1 |  |
| median | 0,1 | 0,1 |  |
| 95%CI | [0,1;0,2] | [0,0;0,1] |  |

^1^U Mann-Whitney

Table Suppl 2. Comparative characteristics of telomeric length in CD8 subpopulations in recipients grouped according to infection status.

|  | **Low risk**  **(n=12)** | **High risk**  **(n=8)** | ***P*-value** |
| --- | --- | --- | --- |
| **tel length** |  |  | 0,5120^1^ |
| mean (SD) | 160,3 (49,6) | 146,3 (47,6) |  |
| range | 79,2-251,5 | 70,1-201,0 |  |
| median | 160,6 | 148,9 |  |
| 95%CI | [128,8;191,9] | [106,5;186,1] |  |
| tel lenght +/- |  |  | 0,1770^1^ |
| mean (SD) | 4,7 (2,3) | 3,4 (1,1) |  |
| range | 2,6-11,1 | 1,6-4,6 |  |
| median | 4,3 | 3,5 |  |
| 95%CI | [3,2;6,1] | [2,5;4,3] |  |
| tel per chromosome |  |  | 0,5892^1^ |
| mean (SD) | 1,7 (0,5) | 1,6 (0,5) |  |
| range | 0,9-2,7 | 0,8-2,2 |  |
| median | 1,8 | 1,6 |  |
| 95%CI | [1,4;2,1] | [1,2;2,0] |  |
| tel +/- per chromosome |  |  | 0,2976^1^ |
| mean (SD) | 0,0 (0,1) | 0,0 (0,0) |  |
| range | 0,0-0,1 | 0,0-0,1 |  |
| median | 0,0 | 0,0 |  |
| 95%CI | [0,0;0,1] | [0,0;0,0] |  |

^1^U Mann-Whitney

Table Suppl 3. Comparative characteristics of telomeric length in CD19 subpopulations in recipients grouped according to infection status.

|  | **Low risk**  **(n=12)** | **High risk**  **(n=8)** | ***P*-value** |
| --- | --- | --- | --- |
| **tel length** |  |  | 0,1770^1^ |
| mean (SD) | 168,3 (82,8) | 217,1 (57,1) |  |
| range | 45,5-296,3 | 129,0-283,6 |  |
| median | 140,7 | 222,7 |  |
| 95%CI | [115,7;220,9] | [169,4;264,8] |  |
| tel lenght +/- |  |  | 0,8471^1^ |
| mean (SD) | 4,9 (2,7) | 5,0 (1,3) |  |
| range | 1,0-10,5 | 3,0-6,5 |  |
| median | 5,0 | 5,1 |  |
| 95%CI | [3,2;6,6] | [3,9;6,1] |  |
| tel per chromosome |  |  | 0,1897^1^ |
| mean (SD) | 1,8 (0,9) | 2,4 (0,6) |  |
| range | 0,5-3,2 | 1,4-3,1 |  |
| median | 1,6 | 2,5 |  |
| 95%CI | [1,3;2,4] | [1,8;2,9] |  |
| tel +/- per chromosome |  |  | 0,7871^1^ |
| mean (SD) | 0,1 (0,1) | 0,1 (0,1) |  |
| range | 0,0-0,1 | 0,0-0,1 |  |
| median | 0,1 | 0,1 |  |
| 95%CI | [0,0;0,1] | [0,0;0,1] |  |

^1^U Mann-Whitney

Table Suppl 4. Comparative characteristics of telomeric length in CD56 subpopulations in recipients grouped according to infection status.

|  | **Low risk**  **(n=12)** | **High risk**  **(n=8)** | ***P*-value** |
| --- | --- | --- | --- |
| **tel length** |  |  | 0,7285^1^ |
| mean (SD) | 169,3 (69,2) | 181,4 (76,8) |  |
| range | 59,9-283,3 | 21,4-269,2 |  |
| median | 183,9 | 190,4 |  |
| 95%CI | [125,4;213,3] | [117,3;245,6] |  |
| tel lenght +/- |  |  | 0,8170^1^ |
| mean (SD) | 7,7 (3,4) | 8,0 (3,8) |  |
| range | 2,8-13,5 | 1,0-12,8 |  |
| median | 8,7 | 8,0 |  |
| 95%CI | [5,5;9,9] | [4,8;11,2] |  |
| tel per chromosome |  |  | 0,7285^1^ |
| mean (SD) | 1,8 (0,7) | 2,0 (0,8) |  |
| range | 0,7-3,1 | 0,2-2,9 |  |
| median | 2,0 | 2,1 |  |
| 95%CI | [1,4;2,3] | [1,3;2,7] |  |
| tel +/- per chromosome |  |  | 0,6713^1^ |
| mean (SD) | 0,1 (0,0) | 0,1 (0,0) |  |
| range | 0,0-0,1 | 0,0-0,1 |  |
| median | 0,1 | 0,1 |  |
| 95%CI | [0,0;0,1] | [0,1;0,1] |  |

^1^U Mann-Whitney

Table Suppl 5. Comparative characteristics of telomeric length in CD4 subpopulation in recipients grouped according to GvHD status (chronic GvH in anamnesis or not).

|  | **GvH**  **(n=9)** | **No GvH**  **(n=11)** | ***P*-value** |
| --- | --- | --- | --- |
| **tel length** |  |  | 0,3348^1^ |
| mean (SD) | 201,0 (134,3) | 159,3 (81,0) |  |
| range | 106,5-516,8 | 74,0-362,1 |  |
| median | 159,0 | 135,8 |  |
| 95%CI | [88,7;313,3] | [107,9;210,8] |  |
| tel lenght +/- |  |  | 0,2976^1^ |
| mean (SD) | 12,6 (10,8) | 9,1 (7,0) |  |
| range | 2,5-37,5 | 2,3-26,3 |  |
| median | 10,1 | 7,7 |  |
| 95%CI | [3,5;21,6] | [4,6;13,5] |  |
| tel per chromosome |  |  | 0,3961^1^ |
| mean (SD) | 2,2 (1,5) | 1,7 (0,9) |  |
| range | 1,2-5,6 | 0,8-3,9 |  |
| median | 1,7 | 1,5 |  |
| 95%CI |  | [1,2;2,3] |  |
| tel +/- per chromosome |  |  | 0,3961^1^ |
| mean (SD) | 0,1 (0,1) | 0,1 (0,1) |  |
| range | 0,0-0,4 | 0,0-0,3 |  |
| median | 0,1 | 0,1 |  |
| 95%CI | [0,0;0,2] | [0,0;0,1] |  |

^1^U Mann-Whitney

Table Suppl 6. Comparative characteristics of telomeric length in CD8 subpopulation in recipients grouped according to GvHD status (chronic GvH in anamnesis or not).

|  | **GvH**  **(n=9)** | **No GvH**  **(n=11)** | ***P*-value** |
| --- | --- | --- | --- |
| **tel length** |  |  | 0,2633^1^ |
| mean (SD) | 138,0 (43,5) | 165,9 (49,5) |  |
| range | 70,1-201,0 | 79,2-251,5 |  |
| median | 133,7 | 176,6 |  |
| 95%CI | [101,7;174,4] | [134,4;197,3] |  |
| tel lenght +/- |  |  | 0,3159^1^ |
| mean (SD) | 4,1 (3,0) | 4,2 (1,1) |  |
| range | 1,6-11,1 | 2,5-5,8 |  |
| median | 3,1 | 4,3 |  |
| 95%CI | [1,6;6,6] | [3,5;4,8] |  |
| tel per chromosome |  |  | 0,2633^1^ |
| mean (SD) | 1,5 (0,5) | 1,8 (0,5) |  |
| range | 0,8-2,2 | 0,9-2,7 |  |
| median | 1,5 | 1,9 |  |
| 95%CI | [1,1;1,9] | [1,5;2,1] |  |
| tel +/- per chromosome |  |  | 0,7871^1^ |
| mean (SD) | 0,0 (0,0) | 0,0 (0,0) |  |
| range | 0,0-0,1 | 0,0-0,1 |  |
| median | 0,0 | 0,0 |  |
| 95%CI | [0,0;0,1] | [0,0;0,1] |  |

^1^U Mann-Whitney

Table Suppl 7. Comparative characteristics of telomeric length in CD19 subpopulation in recipients grouped according to GvHD status (chronic GvH in anamnesis or not).

|  | **GvH**  **(n=9)** | **No GvH**  **(n=11)** | ***P*-value** |
| --- | --- | --- | --- |
| **tel length** |  |  | 0,8471^1^ |
| mean (SD) | 190,9 (80,4) | 185,8 (76,5) |  |
| range | 45,5-275,2 | 87,9-296,3 |  |
| median | 211,9 | 179,7 |  |
| 95%CI | [123,7;258,1] | [137,2;234,3] |  |
| tel lenght +/- |  |  | 0,7871^1^ |
| mean (SD) | 5,3 (2,8) | 4,7 (1,8) |  |
| range | 1,0-10,5 | 2,0-6,9 |  |
| median | 5,7 | 4,6 |  |
| 95%CI | [3,0;7,6] | [3,5;5,8] |  |
| tel per chromosome |  |  | 0,8774^1^ |
| mean (SD) | 2,1 (0,9) | 2,0 (0,8) |  |
| range | 0,5-3,0 | 1,0-3,2 |  |
| median | 2,3 | 2,0 |  |
| 95%CI | [1,4;2,8] | [1,5;2,6] |  |
| tel +/- per chromosome |  |  | 0,6713^1^ |
| mean (SD) | 0,1 (0,1) | 0,1 (0,1) |  |
| range | 0,0-0,1 | 0,0-0,1 |  |
| median | 0,1 | 0,1 |  |
| 95%CI | [0,0;0,1] | [0,0;0,1] |  |

^1^U Mann-Whitney

Table Suppl 8. Comparative characteristics of telomeric length in CD56 subpopulation in recipients grouped according to GvHD status (chronic GvH in anamnesis or not).

|  | **GvH**  **(n=9)** | **No GvH**  **(n=11)** | ***P*-value** |
| --- | --- | --- | --- |
| **tel length** |  |  | 0,5120^1^ |
| mean (SD) | 165,5 (61,0) | 180,0 (78,4) |  |
| range | 66,2-254,4 | 21,4-283,3 |  |
| median | 167,1 | 198,8 |  |
| 95%CI | [114,5;216,4] | [130,2;229,8] |  |
| tel lenght +/- |  |  | 0,6713^1^ |
| mean (SD) | 7,4 (2,7) | 8,1 (4,1) |  |
| range | 4,7-12,1 | 1,0-13,5 |  |
| median | 7,6 | 9,5 |  |
| 95%CI | [5,2;9,7] | [5,5;10,6] |  |
| tel per chromosome |  |  | 0,5371^1^ |
| mean (SD) | 1,8 (0,7) | 2,0 (0,8) |  |
| range | 0,7-2,8 | 0,2-3,1 |  |
| median | 1,9 | 2,2 |  |
| 95%CI | [1,3;2,4] | [1,4;2,5] |  |
| tel +/- per chromosome |  |  | 0,2318^1^ |
| mean (SD) | 0,1 (0,0) | 0,1 (0,0) |  |
| range | 0,1-0,1 | 0,0-0,1 |  |
| median | 0,1 | 0,1 |  |
| 95%CI | [0,0;0,0] | [0,0;0,1] |  |

^1^U Mann-Whitney

Table Suppl 9. Comparison of telomeric length in subpopulation of CD4 in donors (D) when grouped according to gender

|  | **f**  **(n=9)** | **m**  **(n=11)** | **D**  **(n=20)** | ***P*-value** |
| --- | --- | --- | --- | --- |
| **tel length** |  |  |  | 0,2241^2^ |
| avr. (SD) | 343,4 (247,3) | 191,8 (109,7) | 260,0 (195,1) |  |
| range | 124,8-775,2 | 37,5-426,6 | 37,5-775,2 |  |
| median | 228,2 | 204,7 | 205,3 |  |
| 95%CI | [153,3;533,5] | [118,2;265,5] | [168,7;351,3] |  |
| **tel lenght +/-** |  |  |  | 0,1489^2^ |
| avr. (SD) | 22,9 (19,7) | 10,6 (9,0) | 16,1 (15,7) |  |
| range | 3,3-56,2 | 2,1-30,9 | 2,1-56,2 |  |
| median | 14,1 | 6,6 | 10,2 |  |
| 95%CI | [7,7;38,0] | [4,5;16,7] | [8,8;23,4] |  |
| **tel per chromosome** |  |  |  | 0,2390^2^ |
| avr. (SD) | 3,7 (2,7) | 2,1 (1,2) | 2,8 (2,1) |  |
| range | 1,4-8,4 | 0,4-4,6 | 0,4-8,4 |  |
| median | 2,5 | 2,2 | 2,2 |  |
| 95%CI | [1,7;5,8] | [1,3;2,9] | [1,8;3,8] |  |
| **tel +/- per chromosome** |  |  |  | 0,0772^1^ |
| avr. (SD) | 0,3 (0,2) | 0,1 (0,1) | 0,2 (0,2) |  |
| range | 0,0-0,6 | 0,0-0,3 | 0,0-0,6 |  |
| median | 0,2 | 0,1 | 0,1 |  |
| 95%CI | [0,1;0,4] | [0,1;0,2] | [0,1;0,3] |  |

^1^t-Student;^2^U Mann-Whitney

**Table Suppl 10. Comparison of telomeric length in subpopulation of CD8 in donors (D) when grouped according to gender**

|  | **f**  **(n=9)** | **m**  **(n=11)** | **D**  **(n=20)** | ***P*-value** |
| --- | --- | --- | --- | --- |
| **tel length** |  |  |  | 0,5433^2^ |
| avr. (SD) | 229,0 (87,2) | 189,6 (80,9) | 207,3 (83,9) |  |
| range | 158,4-413,2 | 75,1-319,8 | 75,1-413,2 |  |
| median | 194,6 | 195,0 | 194,8 |  |
| 95%CI | [162,0;296,0] | [135,3;243,9] | [168,0;246,6] |  |
| **tel lenght +/-** |  |  |  | 0,6761^2^ |
| avr. (SD) | 5,3 (2,0) | 5,2 (1,9) | 5,2 (1,9) |  |
| range | 3,6-9,5 | 1,9-8,4 | 1,9-9,5 |  |
| median | 4,5 | 5,4 | 4,7 |  |
| 95%CI | [3,7;6,8] | [4,0;6,5] | [4,4;6,1] |  |
| **tel per chromosome** |  |  |  | 0,5184^2^ |
| avr. (SD) | 2,5 (0,9) | 2,1 (0,9) | 2,3 (0,9) |  |
| range | 1,7-4,5 | 0,8-3,5 | 0,8-4,5 |  |
| median | 2,1 | 2,1 | 2,1 |  |
| 95%CI | [1,8;3,2] | [1,5;2,7] | [1,8;2,7] |  |
| **tel +/- per chromosome** |  |  |  | 1,00^2^ |
| avr. (SD) | 0,0 (0,1) | 0,0 (0,1) | 0,0 (0,1) |  |
| range | 0,0-0,1 | 0,0-0,1 | 0,0-0,1 |  |
| median | 0,0 | 0,0 | 0,0 |  |
| 95%CI | [0,0;0,1] | [0,0;0,1] | [0,0;0,1] |  |

^1^t-Student;^2^U Mann-Whitney

**Table Suppl 11. Comparison of telomeric length in subpopulation of CD19 in donors (D) when grouped according to gender**

|  | **f**  **(n=9)** | **m**  **(n=11)** | **D**  **(n=20)** | ***P*-value** |
| --- | --- | --- | --- | --- |
| **tel length** |  |  |  | 0,4793^1^ |
| avr. (SD) | 219,0 (90,3) | 244,5 (67,6) | 233,0 (77,5) |  |
| range | 101,0-358,9 | 148,8-347,5 | 101,0-358,9 |  |
| median | 198,7 | 215,8 | 213,7 |  |
| 95%CI | [149,6;288,4] | [199,1;289,9] | [196,8;269,3] |  |
| **tel lenght +/-** |  |  |  | 0,1599^2^ |
| avr. (SD) | 5,1 (2,1) | 7,7 (4,9) | 6,5 (4,0) |  |
| range | 2,3-8,3 | 3,4-18,3 | 2,3-18,3 |  |
| median | 4,6 | 5,4 | 5,0 |  |
| 95%CI | [3,4;6,7] | [4,5;11,0] | [4,6;8,4] |  |
| **tel per chromosome** |  |  |  | 0,5119^1^ |
| avr. (SD) | 2,4 (1,0) | 2,6 (0,7) | 2,5 (0,8) |  |
| range | 1,1-3,9 | 1,6-3,8 | 1,1-3,9 |  |
| median | 2,2 | 2,3 | 2,3 |  |
| 95%CI | [1,6;3,1] | [2,2;3,1] | [2,1;2,9] |  |
| **tel +/- per chromosome** |  |  |  | 0,1837^2^ |
| avr. (SD) | 0,0 (0,1) | 0,1 (0,1) | 0,1 (0,1) |  |
| range | 0,0-0,1 | 0,0-0,2 | 0,0-0,2 |  |
| median | 0,0 | 0,1 | 0,1 |  |
| 95%CI | [0,0;0,1] | [0,0;0,1] | [0,0;0,1] |  |

^1^t-Student;^2^U Mann-Whitney

Table Suppl 12. Comparison of telomeric length in subpopulation of CD56 in donors (D) when grouped according to gender

|  | **f**  **(n=9)** | **m**  **(n=11)** | **D**  **(n=20)** | ***P*-value** |
| --- | --- | --- | --- | --- |
| **tel length** |  |  |  | 0,2098^1^ |
| avr. (SD) | 202,6 (39,6) | 172,2 (58,9) | 186,6 (51,7) |  |
| range | 134,3-264,3 | 104,3-282,0 | 104,3-282,0 |  |
| median | 198,0 | 173,2 | 182,4 |  |
| 95%CI | [172,2;233,0] | [130,1;214,3] | [161,7;211,5] |  |
| **tel lenght +/-** |  |  |  | 0,4356^1^ |
| avr. (SD) | 9,1 (2,5) | 8,0 (3,2) | 8,5 (2,8) |  |
| range | 4,8-12,6 | 2,4-13,4 | 2,4-13,4 |  |
| median | 9,3 | 8,1 | 8,3 |  |
| 95%CI | [7,2;10,9] | [5,8;10,3] | [7,1;9,9] |  |
| **tel per chromosome** |  |  |  | 0,2127^1^ |
| avr. (SD) | 2,2 (0,4) | 1,9 (0,7) | 2,0 (0,6) |  |
| range | 1,5-2,9 | 1,1-3,1 | 1,1-3,1 |  |
| median | 2,2 | 1,9 | 2,0 |  |
| 95%CI | [1,9;2,5] | [1,4;2,3] | [1,8;2,3] |  |
| **tel +/- per chromosome** |  |  |  | 0,6940^2^ |
| avr. (SD) | 0,1 (0,0) | 0,1 (0,0) | 0,1 (0,0) |  |
| range | 0,1-0,1 | 0,0-0,1 | 0,0-0,1 |  |
| median | 0,1 | 0,1 | 0,1 |  |
| 95%CI | [0,0;0,0] | [0,0;0,1] | [0,1;0,1] |  |

^1^t-Student;^2^U Mann-Whitney

**Table Suppl 13. Comparison of telomeric length in subpopulation of CD4 in recipients (R) when grouped according to gender**

|  | **f**  **(n=8)** | **m**  **(n=12)** | **R**  **(n=20)** | ***P*-value** |
| --- | --- | --- | --- | --- |
| **tel length** |  |  |  | 0,6160^2^ |
| avr. (SD) | 181,9 (138,8) | 172,1 (80,6) | 176,0 (104,3) |  |
| range | 98,0-516,8 | 74,0-362,1 | 74,0-516,8 |  |
| median | 135,8 | 150,0 | 146,9 |  |
| 95%CI | [65,8;297,9] | [120,8;223,3] | [127,2;224,8] |  |
| **tel lenght +/-** |  |  |  | 0,5371^2^ |
| avr. (SD) | 10,7 (11,4) | 10,4 (6,8) | 10,5 (8,6) |  |
| range | 2,3-37,5 | 2,5-26,3 | 2,3-37,5 |  |
| median | 7,8 | 8,8 | 8,2 |  |
| 95%CI | [1,1;20,2] | [6,0;14,7] | [6,4;14,5] |  |
| **tel per chromosome** |  |  |  | 0,6160^2^ |
| avr. (SD) | 2,0 (1,5) | 1,9 (0,9) | 1,9 (1,1) |  |
| range | 1,1-5,6 | 0,8-3,9 | 0,8-5,6 |  |
| median | 1,5 | 1,6 | 1,6 |  |
| 95%CI | [0,7;3,2] | [1,3;2,4] | [1,4;2,4] |  |
| **tel +/- per chromosome** |  |  |  | 0,4179^2^ |
| avr. (SD) | 0,1 (0,1) | 0,1 (0,1) | 0,1 (0,1) |  |
| range | 0,0-0,4 | 0,0-0,3 | 0,0-0,4 |  |
| median | 0,1 | 0,1 | 0,1 |  |
| 95%CI | [0,0;0,2] | [0,1;0,2] | [0,1;0,2] |  |

^1^t-Student;^2^U Mann-Whitney

Table Suppl 14. Comparison of telomeric length in subpopulation of CD8 in recipients (R) when grouped according to gender

|  | **f**  **(n=8)** | **m**  **(n=12)** | **R**  **(n=20)** | ***P*-value** |
| --- | --- | --- | --- | --- |
| **tel length** |  |  |  | 0,6752^1^ |
| avr. (SD) | 149,0 (58,8) | 158,5 (41,9) | 154,7 (48,1) |  |
| range | 70,1-251,5 | 79,2-210,5 | 70,1-251,5 |  |
| median | 136,4 | 160,6 | 152,5 |  |
| 95%CI | [99,9;198,1] | [131,9;185,2] | [132,2;177,2] |  |
| **tel lenght +/-** |  |  |  | 0,1770^2^ |
| avr. (SD) | 3,5 (1,4) | 4,6 (2,2) | 4,1 (2,0) |  |
| range | 1,6-5,8 | 2,6-11,1 | 1,6-11,1 |  |
| median | 3,2 | 4,4 | 4,1 |  |
| 95%CI | [2,3;4,6] | [3,2;6,0] | [3,2;5,1] |  |
| **tel per chromosome** |  |  |  | 0,6172^1^ |
| avr. (SD) | 1,6 (0,6) | 1,7 (0,4) | 1,7 (0,5) |  |
| range | 0,8-2,7 | 0,9-2,3 | 0,8-2,7 |  |
| median | 1,5 | 1,8 | 1,7 |  |
| 95%CI | [1,1;2,1] | [1,4;2,0] | [1,4;1,9] |  |
| **tel +/- per chromosome** |  |  |  | 0,2976^2^ |
| avr. (SD) | 0,0 (0,0) | 0,0 (0,1) | 0,0 (0,0) |  |
| range | 0,0-0,1 | 0,0-0,1 | 0,0-0,1 |  |
| median | 0,0 | 0,0 | 0,0 |  |
| 95%CI | [0,0;0,0] | [0,0;0,1] | [0,0;0,1] |  |

^1^t-Student;^2^U Mann-Whitney

**Table Suppl 15. Comparison of telomeric length in subpopulation of CD19 in recipients (R) when grouped according to gender**

|  | **f**  **(n=8)** | **m**  **(n=12)** | **R**  **(n=20)** | ***P*-value** |
| --- | --- | --- | --- | --- |
| **tel length** |  |  |  | 0,8205^1^ |
| avr. (SD) | 192,2 (52,0) | 184,9 (90,7) | 187,8 (76,0) |  |
| range | 129,0-283,6 | 45,5-296,3 | 45,5-296,3 |  |
| median | 185,9 | 185,5 | 185,9 |  |
| 95%CI | [148,8;235,7] | [127,2;242,5] | [152,2;223,4] |  |
| **tel lenght +/-** |  |  |  | 0,4385^1^ |
| avr. (SD) | 4,4 (1,2) | 5,2 (2,7) | 4,9 (2,2) |  |
| range | 3,0-6,5 | 1,0-10,5 | 1,0-10,5 |  |
| median | 4,3 | 5,9 | 5,0 |  |
| 95%CI | [3,5;5,4] | [3,5;6,9] | [3,9;5,9] |  |
| **tel per chromosome** |  |  |  | 0,8590^1^ |
| avr. (SD) | 2,1 (0,6) | 2,0 (1,0) | 2,1 (0,8) |  |
| range | 1,4-3,1 | 0,5-3,2 | 0,5-3,2 |  |
| median | 2,1 | 2,1 | 2,1 |  |
| 95%CI | [1,6;2,6] | [1,4;2,7] | [1,7;2,4] |  |
| **tel +/- per chromosome** |  |  |  | 0,2976^2^ |
| avr. (SD) | 0,0 (0,1) | 0,1 (0,0) | 0,1 (0,1) |  |
| range | 0,0-0,1 | 0,0-0,1 | 0,0-0,1 |  |
| median | 0,0 | 0,1 | 0,1 |  |
| 95%CI | [0,0;0,1] | [0,0;0,1] | [0,0;0,1] |  |

^1^t-Student;^2^U Mann-Whitney

Table Suppl 16. Comparison of telomeric length in subpopulation of CD56 in recpients (R) when grouped according to gender

|  | **f**  **(n=8)** | **m**  **(n=12)** | **R**  **(n=20)** | ***P*-value** |
| --- | --- | --- | --- | --- |
| **tel length** |  |  |  | 0,4259^1^ |
| avr. (SD) | 158,3 (69,7) | 184,8 (72,1) | 174,2 (70,6) |  |
| range | 21,4-269,2 | 59,9-283,3 | 21,4-283,3 |  |
| median | 167,1 | 207,3 | 183,9 |  |
| 95%CI | [100,0;216,6] | [138,9;230,6] | [141,1;207,2] |  |
| **tel lenght +/-** |  |  |  | 0,3749^2^ |
| avr. (SD) | 7,2 (3,7) | 8,3 (3,5) | 7,8 (3,5) |  |
| range | 1,0-12,8 | 4,0-13,5 | 1,0-13,5 |  |
| median | 8,0 | 9,8 | 8,2 |  |
| 95%CI | [4,1;10,2] | [6,1;10,5] | [6,2;9,5] |  |
| **tel per chromosome** |  |  |  | 0,4204^1^ |
| avr. (SD) | 1,7 (0,8) | 2,0 (0,8) | 1,9 (0,8) |  |
| range | 0,2-2,9 | 0,7-3,1 | 0,2-3,1 |  |
| median | 1,9 | 2,3 | 2,0 |  |
| 95%CI | [1,1;2,4] | [1,5;2,5] | [1,5;2,3] |  |
| **tel +/- per chromosome** |  |  |  | 0,7871^2^ |
| avr. (SD) | 0,1 (0,0) | 0,1 (0,0) | 0,1 (0,0) |  |
| range | 0,0-0,1 | 0,0-0,1 | 0,0-0,1 |  |
| median | 0,1 | 0,1 | 0,1 |  |
| 95%CI | [0,0;0,1] | [0,1;0,1] | [0,1;0,1] |  |

^1^t-Student;^2^U Mann-Whitney

**Table Suppl 17. Analysis of correlation of telomeric length of donors and recipients and age of donors (D)**

|  | **Donors (D)** | | **Recipients(R)** | |
| --- | --- | --- | --- | --- |
|  | **Correlation**  **Coefficient ^1^** | ***P*-value** | **Correlation**  **Coefficient ^1^** | ***P*-value** |
| CD4 |  |  |  |  |
| tel per chromosome | -0,21 | 0,3647 | -0.02 | 0.9206 |
| CD8 |  |  |  |  |
| tel per chromosome | -0,59 | 0,0066 | -0.15 | 0.5387 |
| CD19 |  |  |  |  |
| tel per chromosome | -0,26 | 0,2677 | -0.29 | 0.2189 |
| CD56 |  |  |  |  |
| tel per chromosome | -0,08 | 0,7573 | -0.11 | 0.6312 |
|  |  |  |  |  |

Table Suppl 18. Analysis of correlation of the number of CD34^+^ cells infused and telomeric length of the recipients.

|  | | **Correlation**  **Coefficient ^1^** | | | ***P*-value** | |
| --- | --- | --- | --- | --- | --- | --- |
| **CD4** | |  | | |  | |
| **tel per chromosome** | | -0.32 | | | 0.2925 | |
| **CD8** | |  | | |  | |
| **tel per chromosome** | | -0.55 | | | 0.0492 | |
| **CD19** | |  | | |  | |
| **tel per chromosome** | | -0.07 | | | 0.8161 | |
| **CD56** | |  | | |  | |
| **tel per chromosome** | | -0.39 | | | 0.1863 | |
|  |  | |  |  | |  |

^1^Spearman

Table Suppl 19. Immunophenotype comparison between recipients of allo-HCT grouped according to chronic GvH disease history

| **Parameter** | **cGvHD** | **Without cGvHD** | ***P*-value** |
| --- | --- | --- | --- |
| Treg Helios- Eomes |  |  | **0,0227** |
| mean (SD) | 4.1 (1.3) | 8.7 (4.8) |  |
| range | 2.4-5.4 | 4.2-19.1 |  |
| median | 4.6 | 7.2 |  |
| 95%CI | [2.7;5.5] | [5.2;12.1] |  |
| B1 PD1 |  |  | **0,0147** |
| mean (SD) | 4.0 (2.7) | 10.4 (5.5) |  |
| range | 0.2-8.7 | 3.6-18.7 |  |
| median | 3.7 | 9.7 |  |
| 95%CI | [1.2;6.9] | [6.4;14.3] |  |
| B2 PD1 |  |  | **0,0448** |
| mean (SD) | 0.7 (0.7) | 1.8 (1.8) |  |
| range | 0.1-2.1 | 0.6-6.2 |  |
| median | 0.5 | 1.1 |  |
| CD19 PD1 |  |  | **0,0147** |
| mean (SD) | 1.2 (0.9) | 3.3 (2.3) |  |
| range | 0.2-2.9 | 1.2-8.9 |  |
| median | 0.9 | 3.0 |  |
| 95%CI | [0.2;2.2] | [1.6;4.9] |  |
